# Translational Regulation of the *Drosophila* Post-Translational Circadian Mechanism

**DOI:** 10.1371/journal.pgen.1004628

**Published:** 2014-09-11

**Authors:** Jeffrey L. Price

**Affiliations:** University of Missouri-Kansas City, School of Biological Sciences, Division of Molecular Biology and Biochemistry, Kansas City, Missouri, United States of America; Washington University Medical School, United States of America

**Figure 1:**
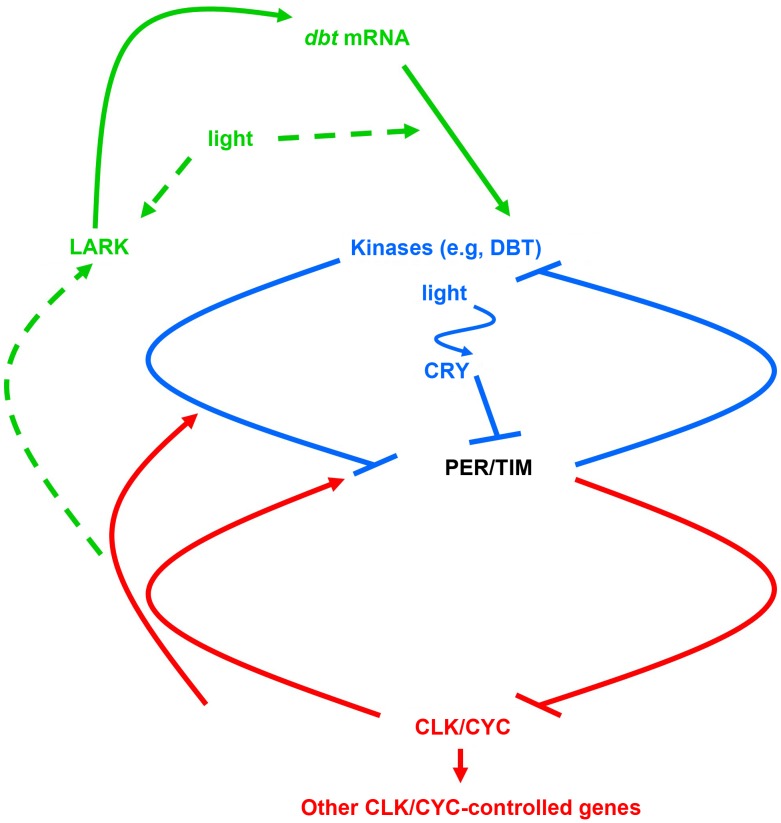
The molecular mechanism for the *Drosophila* circadian clock. This mechanism has been proposed to consist of a transcriptional feedback loop (red) and a post-translational feedback loop (blue). This paper adds a translational regulatory loop (green) in which the LARK protein binds to *dbt* mRNAs to promote translation of DBT, the principal circadian kinase; tentative pathways are marked with dashed arrows.

Much of our behavior and physiology exhibits daily oscillations driven by a circadian rhythm. While the phase of these oscillations is typically set by the daily light–dark cycle, the oscillations themselves are actually produced by an internal circadian clock. A summary of the *Drosophila* circadian clock mechanism, which exhibits evolutionary conservation with the human one, is outlined in Figure 1. Daily oscillations of the transcription factor *period* protein (PER) lead to elevated nuclear PER levels in the late night and early morning, when PER binds to a positively acting CLOCK/CYCLE (CLK/CYC) transcription factor to repress transcription of genes with a CLK/CYC-responsive promoter. One of these genes is the *per* gene, so PER regulates its own transcription in a transcriptional negative feedback loop (colored red in Figure 1). Delays in the negative feedback loop allow *per* mRNA to accumulate to its daily peak and PER protein to persist as a repressor even as its mRNA levels fall. The delays are thought to arise principally from a post-translational feedback loop (colored blue in Figure 1) in which PER is phosphorylated by the casein kinase I ortholog *doubletime* (DBT), resulting in PER degradation throughout the daytime. During the night, PER accumulates because it is no longer degraded in response to light signals (transduced by the CRY photoreceptor and the TIM protein) and represses CLK/CYC-dependent transcription, including that of *per*, *tim*, and many genes leading to the physiological consequences (outputs) of the clock [1]. Now, in this issue of *PLOS Genetics*, Yanmei Huang and coauthors demonstrate translational regulation that in turn regulates the post-translational regulatory loop of the *Drosophila* circadian clock (green loop in Figure 1) [2].

Some earlier work did implicate a role for translational regulation in clock-related processes. For instance, the circadian bioluminescence rhythm in *Gonyaulax* (a single-celled dinoflagellate) has been shown to arise from circadian control of luciferin-binding protein translation [3]. In *Neurospora* (a bread mold), differential translation initiation at two different AUG codons of the circadian protein FRQ extends the effective temperature range for circadian rhythmicity [4]. But there was little evidence that translational control was necessary to support the underlying oscillator mechanism.

However, recently regulation of protein translation has been increasingly moved to a place in the oscillator mechanism itself, as has been reviewed extensively elsewhere [1] and is briefly summarized here. Work in *Drosophila* has identified *per* RNA-binding proteins that stimulate PER translation, demonstrating a role for translational regulation in the transcriptional feedback loop [5], [6]. Work in a number of systems has shown that regulation of translation through circadian changes in polyA tail length [7], Tor signaling [8], [9], ribosome biogenesis [8], and miRNA [1] contributes to the daily oscillations of many proteins whose mRNAs do not oscillate [10], thereby demonstrating a significant role for translational regulation in circadian output pathways [1].

The current work of Huang and coauthors [2] builds on their previous findings that the circadian RNA-binding and translational regulator LARK binds to RNA encoding the circadian kinase DBT [11] and that circadian changes in translation are common for many mRNAs [12]. In the current study, the authors show that LARK binds to each of the four alternatively spliced *dbt* transcripts. By recovering transcripts that co-immunoprecipitate with a tagged ribosomal protein expressed specifically in circadian neurons, it is shown that LARK promotes the translation of these *dbt* mRNAs, because lower levels are associated with ribosomes in the absence of LARK and higher levels with LARK overexpression. Moreover, translation of one of these transcripts undergoes circadian changes in constant darkness (but curiously, not in light–dark cycles), while the translation of another transcript is light inducible with a requirement for LARK (i.e., its translation is not light induced with *lark* knock-down, and the induction by light is increased with LARK overexpression.). Since DBT is involved in setting the circadian period, altered translation of DBT would be predicted to alter circadian period, and in fact, altered LARK abundance does produce changes in circadian period. Knock-down of LARK in the brain neurons that produce rhythms of behavior in constant darkness shortens circadian period, while overexpression of LARK in these neurons lengthens the period. These changes are modified by changes in the *dbt* genotype. For instance, they are not produced in the presence of catalytically inactive DBT, suggesting that the period-altering effects of LARK are mediated through DBT and require DBT activity. Increased expression of LARK delays PER degradation at the beginning of the day in behaviorally relevant brain neurons, suggesting that it reduces DBT-dependent degradation at these times. This might seem counterintuitive, since translation of DBT (which targets PER for degradation) is increased with LARK overexpression. But immunoblot analysis shows that the increased DBT from LARK overexpression comes in a number of atypical molecular weight ranges that include a lower molecular weight form unlikely to retain catalytic activity, as well as some very high molecular weight forms. The authors hypothesize that the lower molecular weight form may interact with full-length DBT to regulate circadian period, so that the ratio of the two determines period. One way this could happen would be if the short isoform and the full-length DBT isoform associate to promote preferential phosphorylation of a PER domain that has previously been shown to slow the pace of the clock rather than accelerate it [13], [14].

The authors present a very creative and provocative model that makes a number of predictions. The short DBT isoform is proposed as a regulator of DBT activity, so it will be important to determine if it is expressed and exhibits circadian oscillations under normal conditions (i.e., without LARK or DBT overexpression, which presumably raises its levels above the current lower limit of detection). Moreover, what exactly are its sequence and role, does it associate with full-length DBT as proposed to target a specific domain in PER and does it interact with another recently described regulator of DBT [15]?

The findings also raise additional questions. Both the levels of the small DBT isoform detected with LARK overexpression and the translation of the light-inducible mRNA exhibit a diurnal oscillation that peaks during the day, and the basis for this oscillation could be the previously demonstrated LARK oscillation, since LARK is also higher during the day than at night. But how does the circadian clock (and/or light) control these oscillations (dashed arrow to LARK in Figure 1)? Moreover, it is not yet known if LARK is acutely induced by light to mediate the effects of light on translation, or alternatively if light induces translation through a LARK-independent pathway, with LARK increasing the magnitude of the effect (dashed arrows from light in Figure 1). It is also not known whether the circadian and diurnal changes in translation occur throughout the circadian system or only in specific circadian cells. Finally, does a similar translational mechanism exist for the mammalian circadian clock, in which mammalian DBT orthologs subserve a similar role [16] and in which a LARK ortholog has also been implicated in circadian translation of PER [17]? If so, the work has revealed an evolutionarily conserved translational mechanism for the regulation of the post-translational loop of the circadian clock.
